# Supplementary material for: Characterization and functional analysis of cathelicidin-MH, a novel frog-derived peptide with anti-septicemic properties
Source: eLife. 2021 Apr 20;10:e64411. doi: 10.7554/eLife.64411 (PMC8057816; doi:10.7554/eLife.64411)
Supplement: Supplementary file 1. [file elife-64411-supp1.docx]

| **Condition^b^** | **Helix**^a^**(%)** | **Parallel**^a^**(%)** | **Turn**^a^**(%)** | **Random**^a^**(%)** | **Total Sum(%)** |
| --- | --- | --- | --- | --- | --- |
| 0 mM SDS | 4.50% | 53.80% | 25.40% | 29.50% | 113.20% |
| 30 mM SDS | 92.30% | 1.40% | 7.50% | 0.80% | 102.00% |
| 60 mM SDS | 92.10% | 1.40% | 7.60% | 0.80% | 101.90% |
| 90 mM SDS | 92.90% | 1.40% | 7.40% | 0.70% | 102.40% |
| 120 mM SDS | 93.80% | 1.40% | 7.00% | 0.70% | 102.80% |
| 0 mM NaCl | 92.40% | 1.40% | 7.70% | 0.80% | 102.30% |
| 100 mM NaCl | 93.70% | 1.30% | 8.80% | 0.40% | 104.20% |
| 200 mM NaCl | 93.30% | 1.40% | 7.80% | 0.60% | 103.10% |
| 400 mM NaCl | 89.60% | 1.40% | 7.60% | 1.10% | 99.70% |
| 20℃ | 94.00% | 1.30% | 7.40% | 0.50% | 103.20% |
| 37℃ | 94.00% | 1.30% | 7.40% | 0.50% | 103.20% |
| 50℃ | 93.50% | 1.30% | 7.30% | 0.60% | 102.70% |
| 70℃ | 94.20% | 1.30% | 7.40% | 0.50% | 103.40% |
| 90℃ | 95.10% | 1.20% | 7.60% | 0.40% | 104.30% |
| LPS | 31.40% | 29.40% | 12.50% | 22.40% | 95.60% |
| LPS + Cath-MH | 90.80% | 1.80% | 7.40% | 1.50% | 101.50% |

**Supplementary file 1.** Secondary structural components of cath-MH in different environments.^a^ CDNN software was used to deconvolute CD spectra into fractional contents and these data are the average value of three scans; ^b^ 213 µg/ml cath-MH was treated with various concentrations of SDS, salt solutions, temperatures, and 50 µM LPS.
