# Supplementary material for: Characterization and functional analysis of cathelicidin-MH, a novel frog-derived peptide with anti-septicemic properties
Source: eLife. 2021 Apr 20;10:e64411. doi: 10.7554/eLife.64411 (PMC8057816; doi:10.7554/eLife.64411)
Supplement: Supplementary file 2. [file elife-64411-supp2.docx]

| **Condition** | **Parameter** |
| --- | --- |
| Plate type | MRC 2-Wells Crystallization UVXPO Microplate (Swissci) |
| Temperature | 277 K |
| Sample concentration | 2.719 mg/ml |
| Peptide solution | TBS buffer pH 7.5 |
| Composition of reservoir solution | 2 M ammonium sulfate |
| Volume and ratio of drop | 2 μl, 1:1 |
| Volume of reservoir | 50 μl |

**Supplementary file 2.** Conditions for growing cath-MH crystals used for X-ray diffraction.
