# Supplementary material for: Characterization and functional analysis of cathelicidin-MH, a novel frog-derived peptide with anti-septicemic properties
Source: eLife. 2021 Apr 20;10:e64411. doi: 10.7554/eLife.64411 (PMC8057816; doi:10.7554/eLife.64411)
Supplement: Supplementary file 3. [file elife-64411-supp3.docx]

| **Condition** | **Parameter** |
| --- | --- |
| Diffraction source | MX 14.2, BESSY II |
| Wavelength (Å) | 0.9184 |
| Temperature (K) | 100 |
| Detector | PILATUS3S 2M |
| Crystal-detector distance (mm) | 251.228 |
| Rotation range per image (°) | 0.10 |
| Total rotation range (°) | 360 |
| Exposure time per image (s) | 0.05 |
| Space group | *R*32 (155) |
| Cell dimensions | |
| a, b, c (Å) | 53.80, 53.80, 64.33 |
| Α, β, γ (°) | 90.00, 90.00, 120.00 |
| Resolution (Å) | 37.73-2.20 (2.26-2.20) |
| Multiplicity | 19.22 (18.64) |
| Number of reflections | |
| Total | 36749 (5500) |
| Unique | 1912 (295) |
| Completeness (%) | 99.30 (98.00) |
| R_maes_* (%) | 6.70 (61.10) |
| I/σ | 28.83 (5.43) |
| Overall B factor from Wilson plot (Å^2^) | 48.73 |
| **Refinement statistics** | |
| Resolution range (Å) | 37.73-2.20 (2.26-2.20) |
| No. of reflections, working set | 1816 (127) |
| R_work_^†^ (%) / R_free_^‡^ (%) | 24.57/27.37 |
| Number of atoms | |
| Protein | 297 |
| Water | 13 |
| Ion | 2 (chloride) |
| Root mean square deviation | |
| Bond lengths (Å) | 0.013 |
| Bond angles (°) | 1.650 |
| Average B factors (Å^2^) Overall | 57.29 |
| Ramachandran plot | |
| Most favored region (%) | 97.30 (36/39) |
| Allowed region (%)  PDB code | 100 (39/39)  7AL0 |

**Supplementary file 3.** Data collection and refinement statistics of X-ray diffraction. *R_meas_ is a redundancy-independent merging R factor. $R_{\mathrm{meas}}=\sum_{hkl} \left\{ {N\left( hkl \right)}/\left[ N\left( hkl \right)-1 \right] \right\}^{1/2}\sum_{i} \left| I_{i}\left( hkl \right)-\left\langle I\left( hkl \right) \right\rangle\right|/{\sum_{hkl} \sum_{i} I_{i}}\left( hkl \right)$, where $\left\langle I\left( hkl \right) \right\rangle$ is the mean of the $N\left( hkl \right)$ individual measurements $I_{i}\left( hkl \right)$ of the density of reflections$hkl$^†^R_work_= ΣǀǀF_o_ǀ - kǀ F_c_ǀǀ/Σ/F_O_ ǀ, where F_o_ and F_c_ are the observed and calculated structure factors, respectively. ^‡^R_free_ = defined by Brunger (***Brunger, 1992***).
