# Supplementary material for: Characterization and functional analysis of cathelicidin-MH, a novel frog-derived peptide with anti-septicemic properties
Source: eLife. 2021 Apr 20;10:e64411. doi: 10.7554/eLife.64411 (PMC8057816; doi:10.7554/eLife.64411)
Supplement: Supplementary file 4. [file elife-64411-supp4.docx]

| **Thermodynamic parameters** | **Cath-MH** | |
| --- | --- | --- |
|  | **LPS** | **D-(+)-galacturonic acid** |
| **N** | 4.90 ± 1.07 | 3.30 ± 2.4e-2 |
| **∆H (kcal mol^-1^)** | -1.76 ± 0.927 | -3.01 ± 4.7e-2 |
| **∆S (cal mol^-1^deg^-1^)** | 27.35 | 25.38 |
| **T∆S (kcal mol^-1^)** | 5.42 | 5.03 |
| **∆G (kcal mol^-1^)** | -7.17 | -8.04 |
| **K_d_ (µM)** | 5.53 ± 7.27 | 1.29 ± 0.144 |

**Supplementary file 4.** Different thermodynamic parameters from ITC. Thermodynamic parameters derived from the ITC experiment for the interaction of cath-MH with LPS and D-(+)-galacturonic acid in Figure 4. Data are mean ± SEM (n =2 per group).
