# Supplementary material for: Characterization and functional analysis of cathelicidin-MH, a novel frog-derived peptide with anti-septicemic properties
Source: eLife. 2021 Apr 20;10:e64411. doi: 10.7554/eLife.64411 (PMC8057816; doi:10.7554/eLife.64411)
Supplement: Supplementary file 5. [file elife-64411-supp5.docx]

| **Enzyme** | **Concentration (nM)** | **Buffer** | **pH** |
| --- | --- | --- | --- |
| Elastase | 0.1 | 50 mM Hepes buffer, 100 mM NaCl, 0.01% Triton X-100 | 7.4 |
| Chymase | 1 |  |  |
| Trypsin I/III | 0.01 | 50 mM Tris-HCl, 150 mM NaCl, 20 mM CaCl_2_, 0.01% Triton X-100 | 8.0 |
| FXIa | 2000 |  |  |
| FXIIa | 1.2 |  |  |
| Thrombin | 50 | 50 mM Tris-HCl, 150 mM NaCl, 0.01% Triton X-100 | 8.0 |
| β-tryptase | 0.3 | 50 mM Tris-HCl, 50 mM NaCl, 0.05% Triton X-100 | 8.0 |
| Kallikrein | 8 | 20 mM Tris–HCl, 150 mM NaCl, 0.02% Triton X-100 | 8.5 |
| Plasmin | 0.25 |  |  |
| FXa | 0.01 | 20 mM Tris-HCl, 200 mM NaCl, 5 mM CaCl_2_, 0.1% BSA | 8.0 |
| tPA | 10 | 20 mM Tris-HCl, 0.05% Triton X-100 | 8.5 |

**Supplementary file 1E.** The buffers and the enzyme concentrations used in protease inhibition assays (n = 3 per group).
