# Supplementary material for: Characterization and functional analysis of cathelicidin-MH, a novel frog-derived peptide with anti-septicemic properties
Source: eLife. 2021 Apr 20;10:e64411. doi: 10.7554/eLife.64411 (PMC8057816; doi:10.7554/eLife.64411)
Supplement: Supplementary file 6. [file elife-64411-supp6.docx]

| **Name** | **5’primer** | **3’ primer** |
| --- | --- | --- |
| ***TNF-α*** | 5’ -CGGTGCCTATGTCTCAGCCT- 3’ | 5’ –GAGGGTCTGGGCCATAGAAC-3’ |
| ***IL-1β*** | 5’ -ATGGCAACTGTTCCTGAACTC3’ | 5’- GCCCATACTTTAGGAAGACA-3’ |
| ***IL-6*** | 5’- AGTTGCCTTCTTGGGACTGA-3’ | 5’- TCCACGATTTCCCAGAGAAC-3’ |
| ***GAPDH*** | 5’ –GTGAAGGTCGGTGTGAACGGATT-3’ | 5’- GGAGATGATGACCCTTTTGGCTC-3’ |

**Supplementary file 1F.** Primers (mouse) were used for qRT-PCR
